# Dr. Franklin Morris Dixon

**Published:** 1893-07

**Authors:** 


### Obituary.

### DR. FRANKLIN MORRIS DIXON.

Dr. Dixon died June 4, 1893, in the seventy-fifth year of his age.

The most prominent feature in the life of Dr. Dixon was the love he had for his family and his profession and the exceeding care with which every operation, whether of minor or major importance, was performed. He acted in his professional work upon the principle that if it were worth doing at all it was worth doing well. With this as his guiding star, he was recognized by his circle of professional friends as one of the most skilful and artistic gold-foil manipulators in the profession.

Dr. Dixon was born in Petersburg, Huntingdon County, Pennsylvania, April 15, 1819; his parents were James and Ann I. Dixon; he was the sixth of ten children, only two of whom survive him. In 1842, at the age of twenty-three, he entered as a student with Dr. Elisha Neall, of Philadelphia, and after spending one year in the laboratory of this preceptor, learning to carve teeth and swedge plates, he opened his office for the practice of dentistry, which he pursued until the close of his earthly career, June 4, 1893, making the period fifty years of continued practice, most of the time in Philadelphia, though a few years were spent in Pottsville, Pennsylvania. No man had a more loyal patronage. His life and labors are of interest to the present generation of dentists from the fact that he entered the profession at a time when every dentist not only made his own plates, but also manufactured the teeth used in the construction of the denture.

When the writer entered his laboratory, in October, 1851, for the purpose of instruction, the first six months were spent in preparing all the different ingredients used in the manufacture of porcelain teeth—single and block, plain and gum—from the crude material; and in nothing was the character of the subject of this sketch more forcibly manifested than the care with which these materials were selected and ground by hand, with mortar and pestle, and placed in separate jars with fused specimens of the body, and the great variety of blue and yellow enamels, cemented on each, designating the quality and color of contents. The gum color used was made from purple powder of cassius, which itself had to be previously prepared from protochloride of tin and solution of gold, a process which, under his care and instruction, was worked out with most satisfactory results. During over two years of the writer's pupilage, not a block or single tooth—plain, pivot, or gum—was inserted that was not carved in his laboratory with knife in hand,—biscuited, enamelled, and fused in a muffled furnace prepared for the purpose.

Operative dentistry at this period had not made the acquaintance of rubber dam, annealed gold-foil, hand, electric, or automatic mallet, but with napkins, serrated instruments, and hand-pressure, Abbey's and Morgan's soft gold-foil was, in Dr. Dixon's hands, used in performing contour-work as efficiently and as beautifully as any inserted at the present day; not as expeditiously or with the same ease, it is true, but it was accomplished. Never will the writer forget the restoration of an inferior bicuspid which had been destroyed with decay from the labial gum margin, and which, with only napkins to protect the operation from moisture, was in this manner completely contoured. This same indomitable perseverance characterized Dr. Dixon's work as long as he was able to stand by his chair.

Dr. Dixon married, June 1, 1858, Miss Elizabeth Alter, daughter of Solomon Alter, of Philadelphia, a most estimable woman. Four daughters with their mother survive him. Knowing the value of education, every nerve was strained to give his children the most favorable opportunities available.

A generous heart, a free hand, a sanguine temperament, and a restless disposition gave Dr. Dixon many anxious hours, but also endeared to him many friends, who will long remember the enthusiasm and the love he gave his profession.

